# Helpful Suggestions for Every-Day Practice*Read at January, 1917, meeting of Tri-County Dental Society.

**Published:** 1917-06

**Authors:** Arthur H. Merritt

**Affiliations:** New York


### HELPFUL SUGGESTIONS FOR EVERY-DAY PRACTICE*

BY ARTHUR H. MERRITT, D.D.S., NEW YORK

The criticism often heard of many of the papers which are read before dental societies is that they are too technical, that however interesting they may be in theory, they are of little practical value to the busy dentist. There is, no doubt, often grounds for such criticism.

* Read at January, 1917, meeting of Tri-County Dental Society.

For that reason I shall attempt to give you something out of my experience which I have found helpful in making the day's work easier, and which has at the same time often contributed to the success of my work, and the satisfaction of my patients.

I will first call attention to a set of six instruments which I have found exceedingly helpful in the operation of cleansing teeth. They are all modifications of a single instrument, one with a short angle for universal use, with two more of same angle made in rights and lefts. Then another for universal use, with larger angle, and again rights and lefts of this instrument. They cut on both sides, may be used in a pull or push stroke, and when new, cut on the end as does a hoe instrument. They are designed for the removal of salivary tartar, and the serumal deposits which so often fill the subgingival space. There is no place in the mouth which can not be reached with one of these instruments. They should be kept sharp (which is easily done) and as a result are constantly being worn thinner, which is desirable for reaching between teeth where the approximal spaces are narrow. There is probably no operation in the mouth more often carelessly done than is the cleansing of the teeth. One of the reasons for this is that there are few instruments really adapted to the requirements of such an operation. Here are six instruments with which anyone can easily learn to perform this important and much needed operation to the complete satisfaction of everyone concerned. They have been adopted by the New York School of Dental Hygiene for the use of its students. They can be obtained of Charles Grafrath, 360 West 50th Street, for less than fifty cents each. They are made from my own designs.

### A CAVITY VARNISH FOR TEETH SENSITIVE TO THERMAL SHOCK

There is a great variation in the sensitiveness of teeth to temperature changes. The range is so narrow in some cases as to make the use of cold water as a mouth wash decidedly unpleasant. This variation is frequently observed in the preparation of teeth for filling.

A cavity in a tooth may be comparatively superficial and yet so sensitive to thermal shock that the slightest blast from the chip blower will cause pain. The danger of metallic fillings in such teeth is well known. There is also the relatively deep curious cavity, where some protection to the pulp is obvious, yet where for some reason a cement lining is not desirable. A great boon in such cases is a cavity varnish which will protect the pulp against thermal shock, and at the same time make possible the use of any filling material that may be indicated. A preparation which I have found exceedingly valuable in such cases, is one composed of gum damar, commercial zinc oxid and Canada balsam. This may be prepared by anyone in the following manner: Gum damar, 1 dram; zinc oxid, 1 dram; Canada balsam, 5 drops. Fuse and pour into shallow moulds. This is best done by first melting the gum damar and stirring in the zinc oxid and Canada balsam. It is then transferred to a container and allowed to cool when it is ready for use. In appearance, it is hard, white and lustrous. A pellet of cotton saturated with chloroform is rubbed on the surface, which exercises a solvent action on the preparation. When thoroughly saturated, it is transferred to the prepared tooth cavity, the surface of which is coated with the varnish. If a heavy coat is desired, a second application may be made, having evaporated the chloroform from the first application. The edges are then cleaned and the tooth is ready to proceed with the filling. The surface of a cavity so treated will have a white and glistening appearance. Used in this way, it is almost absolute as a nonconductor of heat and cold. A tooth so sensitive to temperature changes that the slightest blast from the chip blower will cause exquisite pain, will be wholly insensible to prolonged blasts once the cavity is thoroughly covered. Usually one application is all that is necessary. It is also exceedingly useful in protecting sensitive dentin against the irritating influence of the acid in cement fillings. In the oxy-phosphate of copper, this irritating effect is often marked, causing no little pain at times of insertion. This will be wholly prevented by first applying the varnish. Not infrequently patients present complaining that certain teeth are sensitive to heat and cold. An examination reveals nothing more serious than a slight abrasion or erosion at the cervix which has not even penetrated through the enamel. On testing, such teeth prove to have slightly hyperemic pulps, and where several are involved, may produce a mild neuralgia of uncertain location. If not corrected, this is a not uncommon sequella. Conditions such as this are usually temporary, often produced by the irritating effects of fruit acids, especially grapes. Excellent results may be obtained by washing off such teeth with warm alcohol, thoroughly drying and painting the involved surface with the varnish, usually making two or three applications. Evaporate the chloroform before allowing the moisture to come in contact with it. With a little care on the part of the patient in brushing these surfaces, an application such as this will often last two or three weeks, protecting the tooth against thermal shock and permitting the capillaries of the pulp to regain their normal tone. Often no other treatment is required. It not infrequently happens that this condition is complicated by exquisite sensitiveness to touch, such as might be produced by the tooth brush, finger nail or a steel instrument. This may be cured by isolating the tooth, preferably with the rubber dam, and applying forty per cent. formaldehyde. The technic I have found most satisfactory is to transfer a small quantity of the formaldehyde to a small receptacle into which is dipped an orange wood stick prepared as for tooth polishing, which is then held over the flame of an alcohol lamp until hot, and then applying it to the irritated surface and vigorously rubbing it back and forth. This is repeated several times, often continuing the treatment for ten or fifteen minutes. When carefully done the results are most satisfactory and often permanent. Before removing the dam or napkin, dry and apply the varnish as directed above. This annoying and often complicated situation may be completely cleared up by the treatment here outlined to the great satisfaction of patient and dentist.

### AN INEXPENSIVE AND QUICKLY MADE TEMPORARY STOPPING

Many dentists are familiar with the fact that eugenol and commercial zinc oxid when properly combined make an excellent temporary stopping, but relatively few realize than even better results can be obtained by substituting oil of cloves for the eugenol, and fewer still realize what a wide range of usefulness it has in every-day dental practice. Nor do they realize how long it will last when properly mixed and legitimately used. It should be mixed with a stiff spatula on a ground glass slab—never on a smooth, glassy surface. The zinc oxid should be added slowly and spatulated until thoroughly incorporated. It is surprising how much can be added to the mass when saturation seems complete. Success depends upon incorporating all the liquid will take up. Its range of usefulness is very wide. Its sedative effect makes it most useful as a temporary stopping in aching teeth with vital pulps, especially in those cases in which there is not an actual exposure, and where time does not permit of doing more than is necessary to afford relief until appointment for treatment can be made. The fact that the cavity does not need to be dry adds to its usefulness. It may also be relied upon to seal medicaments in a tooth, provided it is not subjected to direct pressure from occluding teeth. When applied to cavities on approximal surfaces or on the buccal surfaces of molars, it will last for months, completely protecting the tooth. It should not, of course, be used on the occlusal surfaces of teeth. It requires several hours for it to set, but it never becomes so hard that it can not be easily removed with a hand instrument. Its sedative effect on sensitive dentin is marked; it is cleanly and not unpleasant to the taste, and may be depended upon to last for weeks or months when properly employed.

### OCCLUSAL TRAUMA

In the treatment of pyorrhea there are three things which are fundamental: good root surgery, the establishment of a high order of mouth hygiene which must be maintained by the patient, and the correction of occlusal trauma. The latter, which is absolutely essential to success, is most frequently overlooked. A general and exceedingly vague notion prevails that here and there will be found cases in which it is necessary to grind the occlusal surface of some tooth to protect it against overstress, but relatively few at present realize that practically every case of pyorrhea has as one of its etiological factors, occlusal maladjustment, or what has technically come to be known as occlusal trauma. A condition such as this is quite as likely to occur in mouths with normal occlusion according to the Angle classification. as in mouths in which the occlusion is not normal. This seems to be due to the fact that the occlusal planes of the teeth are not worn sufficiently in the act of mastication to admit of a certain amount of motion or play upon these inclined planes, with the result that the strain which should be dissipated by such motion is communicated to the roots themselves, actually injuring the bony support of the teeth. Just what the changes are that take place in the alveolar process is at present uncertain, but it would seem to be not unlike osteoclasis—a progressive resorption of the bone, which if not corrected will result in the eventual loss of the teeth, no matter what treatment is given the case. While there are cases which can not be corrected without the cooperation of the orthodontist, most of them can be corrected by judicious grinding. This does not mean the indiscriminate grinding which is sometimes practiced, but the careful adjustment of the occlusal planes and incisal edges of teeth as is shown to be necessary by thin carbon paper. This is best done by small stones of assorted sizes and shapes as made by Miller. This should be continued until there is a free play of teeth upon one another without undue stress upon any of them as shown by the carbon paper, and the motion of the tooth as felt with the finger placed lightly upon it. This is not done to relieve a tooth from its normal amount of stress, but from the trauma produced by ill directed force. This is so important to the success of any treatment that it should be given first consideration in every case. Eventual failure is inevitable in most cases in which it is not observed.

### LOCAL ANESTHETICS IN THE TREATMENT OF PYORRHEA

Despite the fact that the gums are abundantly supplied with nerves, they are normally insensitive to pain. Nevertheless, they may under provocation become exquisitely sensitive. This happens not infrequently in cases of pyorrhea with deep pockets, making good root surgery well nigh impossible. Very satisfactory results may be obtained in such cases by the use of dentalone or phenandyne. Dentalone is made by Parke, Davis & Co., and is composed of chlorotone, oil of clove and gaulthera. It has a pleasant odor and not unpleasant taste. Phenandyne is made by Schieffelin & Co., and is a nonescharotic phenol preparation. It is not as pleasant in odor or taste as dentalone, but will often give results in cases where dentalone fails. In using either, transfer a few drops to a suitable receptacle, and where the pocket is deep enough to permit, carry a small pledget of cotton saturated with the solution into the pocket and allow it to remain for a few minutes. During operation on the tooth, dip instrument in the solution from time to time and carry it into the pocket. There are very few cases in which one or the other of these does not give very satisfactory results. I make use of them in every case of advanced pyorrhea which I treat. They are both highly antiseptic, which is always desirable in these cases.

### TOOTH BRUSHES

There are few people who keep their mouths as clean as they should, or as clean as they think they do. There are several reasons for this, but one of them is usually a poor tooth brush. An efficient operation is impossible with inefficient tools, therefore the first step in correcting this condition is to provide the patient with a tooth brush adapted to his needs. The brushes upon the market are legion, but most of them are unsatisfactory, ill shaped and of poor quality. In order to meet this condition in my practice, I have designed and had my druggist make a tooth brush which has proven satisfactory to me and my patients. These brushes are of good quality, of small size, and when properly used will do all that can be expected of any brush in keeping the mouth clean. They are made abroad, and are distributed by my druggist, who delivers them to my patients anywhere in Manhattan without extra expense. They retail at 35 cents each and are made in three grades, S, M and H. Any profit there may be in their sale goes to him. These brushes I prescribe to all my patients, carefully instruct them in their use, often giving them a demonstration in my own mouth. The results have been most gratifying. This is not alone due to the fact that I have placed in their hands a small brush of good quality, but because they have been impressed with the importance of a clean mouth, and once they have learned what that means in comfort to themselves, very few of them lapse back into the old hit-and-miss habit. There is no patent or name upon them, and anyone is free to adopt them who cares to do so.

### ARTERY FORCEPS

An instrument which I have found exceedingly useful in routine practice is a pair of artery forceps, though I do not use them for the purpose for which they were designed. It is an instrument about six inches long, so fashioned that when the beaks are brought together, they may be locked, and anything grasped by them can not be pulled out. Occasionally a patient presents with a quill of the tooth brush or a piece of silk caught between the teeth in such a way that it can not be grasped by the fingers, and so wedged that the cotton pliers can not dislodge it. In such cases the artery forceps is a real blessing. Also for grasping and removing the ligatures used in tying the rubber dam, the braided silk now so generally used for wedging teeth, the little wire bristles placed in roots before X-raying, etc. It may not be needed for any of these uses once a week, but there always comes a time when it is worth its weight in gold. It can be had of any surgical supply house at a trifling cost.

### AN X-RAY OUTFIT FOR THE BUSY DENTIST

Every dentist has come to realize, in part at least, the great value of the X-ray in dental practice, and are availing themselves of its use in certain cases. But relatively few realize that it is indispensable in every-day practice, that this need can not be met by the specialist to whom we may refer our patients, or that an instrument is now available which is almost fool proof, and which anyone can learn to use in such a way as to meet every requirement of the most exacting practice. I refer to the Waugh Unit. This instrument is made by Waite & Bartlett of New York, costs $350, with five per cent. discount for cash, and can be used on a direct or alternating current. It is twelve inches square, forty-six inches high, not including the tube stand which is attached to the top. It is an attractive piece of furniture, made in oak or mahogany, and placed on casters to facilitate its movement to any position about the office. A dark room can be extemporized in any dental office and the office assistant taught in fifteen minutes to do the developing.

After the machine has been installed, the actual cost per picture is less than ten cents. I have two of these machines in my office, and have found them so useful that I would not part with them under any circumstances.

My experience has proven that it is impossible to do good root work without their aid. In fact, it is not too much to say that it is impossible to do good dentistry in all its branches without it. They have been so perfected that no one need be without one.

